# The growth of a xenograft breast cancer tumor model with engineered hyaluronan-accumulating stroma is dependent on hyaluronan and independent of CD44

**DOI:** 10.18632/oncotarget.27302

**Published:** 2019-11-12

**Authors:** Chunmei Zhao, Benjamin J. Thompson, Kelly Chen, Feng Gao, Barbara Blouw, Mathieu Marella, Susan Zimmerman, Trevor Kimbler, Sheryl Garrovillo, Jesse Bahn, Lei Huang, Zhongdong Huang, H. Michael Shepard, Sanna Rosengren, Christopher D. Thanos, Daniel C. Maneval

**Affiliations:** ^1^Halozyme Therapeutics, Inc., San Diego, CA, 92121, USA; ^*^Formerly of Halozyme Therapeutics, Inc., San Diego, CA, 92121, USA

**Keywords:** hyaluronan, breast cancer, xenograft, tumor microenvironment, CD44

## Abstract

Hyaluronan accumulation in the tumor microenvironment is associated with poor prognosis in several solid human cancers. To understand the role of stromal hyaluronan in tumor progression, we engineered 3T3HAS3, a hyaluronan-producing fibroblast cell line, by lentiviral transduction of Balb/c 3T3 cells with the *human hyaluronan synthase 3* *(HAS3)* gene. 3T3HAS3 cells significantly enhanced tumor growth when co-grafted with MDA-MB-468 cells in nude mice. Immunohistochemical analysis of the xenograft tumors showed that MDA-MB-468 cells were surrounded by hyaluronan-accumulating stroma, closely resembling the morphology observed in human breast cancer specimens. Tumor growth of MDA-MB-468 + 3T3HAS3 co-grafts was greatly reduced upon hyaluronan degradation by lentiviral transduction of a human hyaluronidase gene in 3T3HAS3 cells, or by systemic administration of pegvorhyaluronidase alfa (PEGPH20). In contrast, the growth of the co-graft tumors was not inhibited when CD44 expression was reduced or ablated by small hairpin RNA-mediated CD44 knockdown in MDA-MB-468 cells, CD44 CRISPR knockout in 3T3HAS3 cells, or by grafting these cells in CD44 knockout nude mice. Collectively, these data demonstrate that tumor growth of an engineered xenograft breast cancer model with hyaluronan-accumulating stroma can be dependent on hyaluronan and independent of CD44.

## Introduction

The tumor microenvironment (TME) plays an important role in tumor progression, and this observation has stimulated the investigation of therapeutic modalities that target or harness components of the TME, in combination with direct cancer cell targeting. In addition to the exciting progress in cancer immunotherapy, which modulates the immune cells in the TME, several approaches that target the extracellular matrix (ECM) and the stromal fibroblasts have shown promising results in mouse models. Among these are the remodeling of the tumor stroma by “Vitamin D priming”, targeting fibroblast activation protein α (FAP)-expressing fibroblasts, and the focus of this study, targeting hyaluronan (HA) in the TME [1–6].

HA is a component of the ECM in both normal and malignant tissue [7, 8]. It consists of repeating disaccharides of *D*-glucuronic acid and *N*-acetyl-*D*-glucosamine linked by ß-glycosidic bonds. The biological functions of HA are complex due to the heterogeneity in polymer length/molecular weight and, therefore, its physical and biochemical properties [7–9]. High molecular weight (HMW) HA is known to associate with water molecules, and HA accumulation in the TME leads to increased tumor interstitial pressure and vascular compression, and presents a physical barrier to the access of therapeutic agents [1, 2, 10, 11]. In addition, HA functions by engaging various HA binding proteins as well as cellular receptors. Binding of HA to different cellular receptors may modulate a variety of intracellular signaling activities that regulate proliferation, migration, and angiogenesis in the TME [7]. CD44 is the best-characterized receptor for HA and is itself a marker for cancer stem cells. CD44 is a transmembrane glycoprotein receptor that was originally identified to function in leukocyte adhesion and recirculation. HA signaling through CD44 regulates downstream pathways such as the mitogen-activated protein kinase (MAPK) and the phosphoinositide-3-kinase (PI3K) pathways [12]. However, the role of HA-CD44 signaling in tumor progression has not been thoroughly investigated *in vivo*.

HA has been identified as an unfavorable prognostic factor in many cancer types [13–25]. HA can be either associated with malignant tumor cells, the tumor stroma, or both. In fact, high levels of HA were found to be prominent in the stroma in a number of malignancies, including ovarian, non-small-cell lung, thyroid, endometrial, esophagus, stomach, and colon cancers [14–20]. In breast cancer, intense HA staining in peritumoral stroma and the presence of cancer cell-associated HA both predict poor patient survival [21] and are associated with an increased frequency of relapse [26].

Due to its role in the TME, HA has received renewed interest as a therapeutic target in recent years [27–29]. Enzymatic degradation of HA by pegvorhyaluronidase alfa (PEGPH20; PEGylated recombinant human hyaluronidase PH20) can reverse the effects of HA on tumor interstitial pressure and vascular compression, facilitate the access of anti-cancer drugs, and increase anti-tumor efficacy in preclinical models [1–3]. PEGPH20 is currently in clinical development and is being evaluated in a number of clinical trials, including a randomized phase III clinical trial (NCT02715804) in combination with nab-paclitaxel and gemcitabine in patients with stage IV pancreatic ductal adenocarcinoma [30, 31].

HA is synthesized by 3 HA synthase (HAS) enzymes in mammals, HAS1, HAS2, and HAS3, which can form homo- or hetero-dimers. Among the homodimers, HAS3 has the highest enzymatic activity, followed by HAS2 and, finally, HAS1 [32]. In breast cancer, stromal expression of all HAS isoforms was associated with poor patient outcomes [33]. Overexpression of HAS2 and HAS3 has been used to model HA-accumulating tumors and was shown to increase tumor growth [34–37].

In this study, we established a xenograft model in which human triple negative breast cancer MDA-MB-468 cells were pre-mixed with 3T3HAS3 – Balb/c 3T3 cells that were engineered to express the human HAS3 protein and synthesize HA. MDA-MB-468 cells are known to express CD44 and display chemotactic migration toward HMW HA [38]. Using this model, the dependence of tumor growth on HA and CD44 was investigated. While the tumor growth in this xenograft model was clearly dependent on the HA synthesized by the engineered stromal cells, there was no measurable effect when CD44 expression was abolished in tumor cells, the stromal cells, or the host.

## Results

### Engineered 3T3HAS3 fibroblast cells synthesize HMW HA that binds to MDA-MB-468 tumor cells

**Figure 1:**
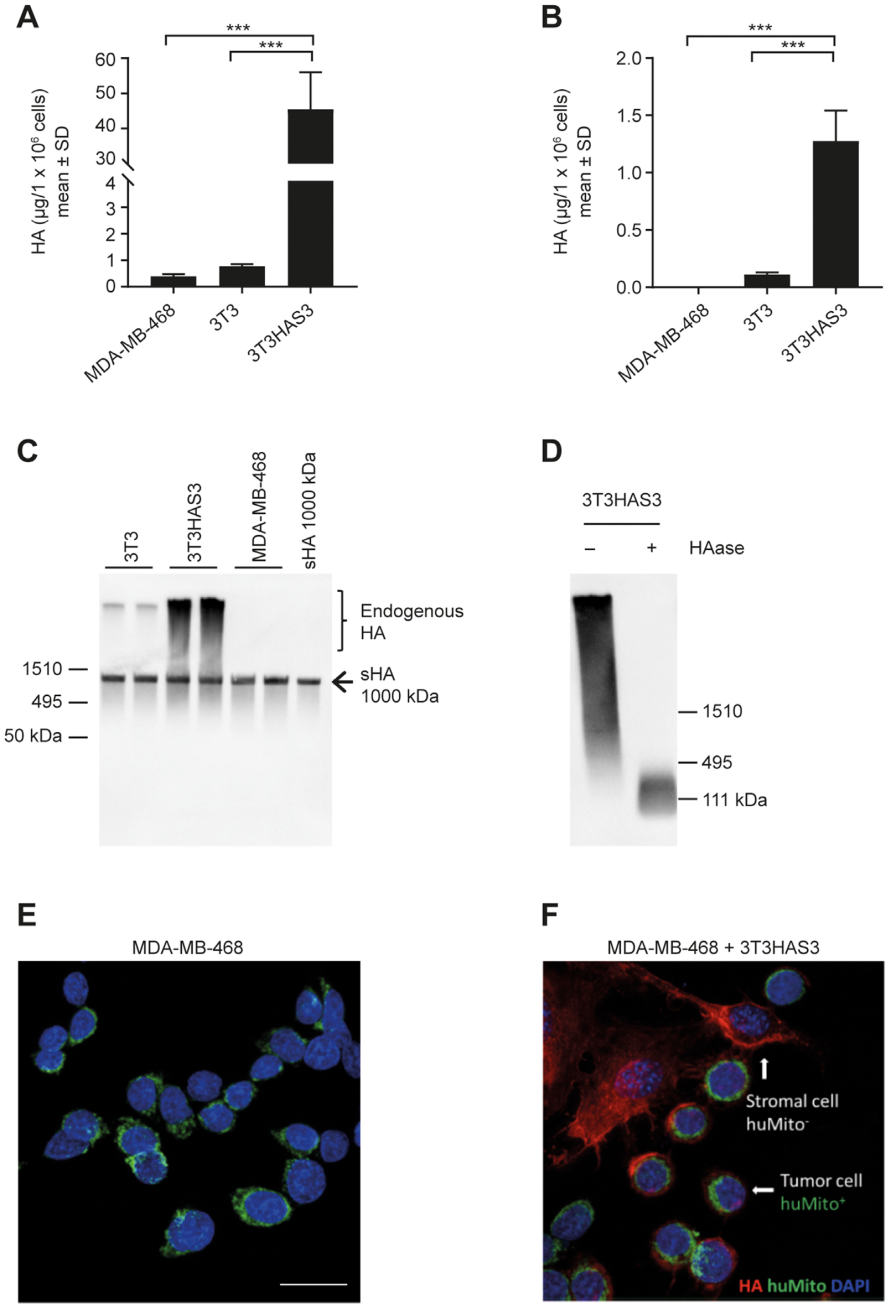
Engineered HA-accumulating fibroblast cells synthesized HMW HA that bound to MDA-MB-468 breast cancer cells in culture. (**A, B**) Engineered 3T3HAS3 cells synthesized increased levels of HA compared with 3T3 and MDA-MB-468 cells, as determined by HA quantification in both culture supernatant (A) and cell pellet (B). (**C**) 3T3HAS3 cells synthesized HMW HA as determined by HA size analysis (HA blot). The arrow indicates the position of Select-HA™ (sHA, 1000 kDa) that was spiked into the samples prior to sample processing. (**D**) HMW HA synthesized by 3T3HAS3 cells was subject to degradation by HAase. (**E**) MDA-MB-468 cells were not associated with HA when cultured alone. MDA-MB-468 cells were identified by staining for human mitochondria (green, huMito+). (**F**) MDA-MB-468 cells were associated with pericellular HA (red) when co-cultured with 3T3HAS3 fibroblast cells (negative for HuMito, HuMito−). Blue: DAPI, green: HuMito, red: HA. Scale bar: 20 μm (E, F). ^***^ *p* < 0.001 (Tukey’s post-test). Abbreviations: DAPI, 4′,6-diamidino-2-phenylindole; HAase, hyaluronidase; HMW, high molecular weight; SD, standard deviation; sHA, Select HA.

**Figure 2:**
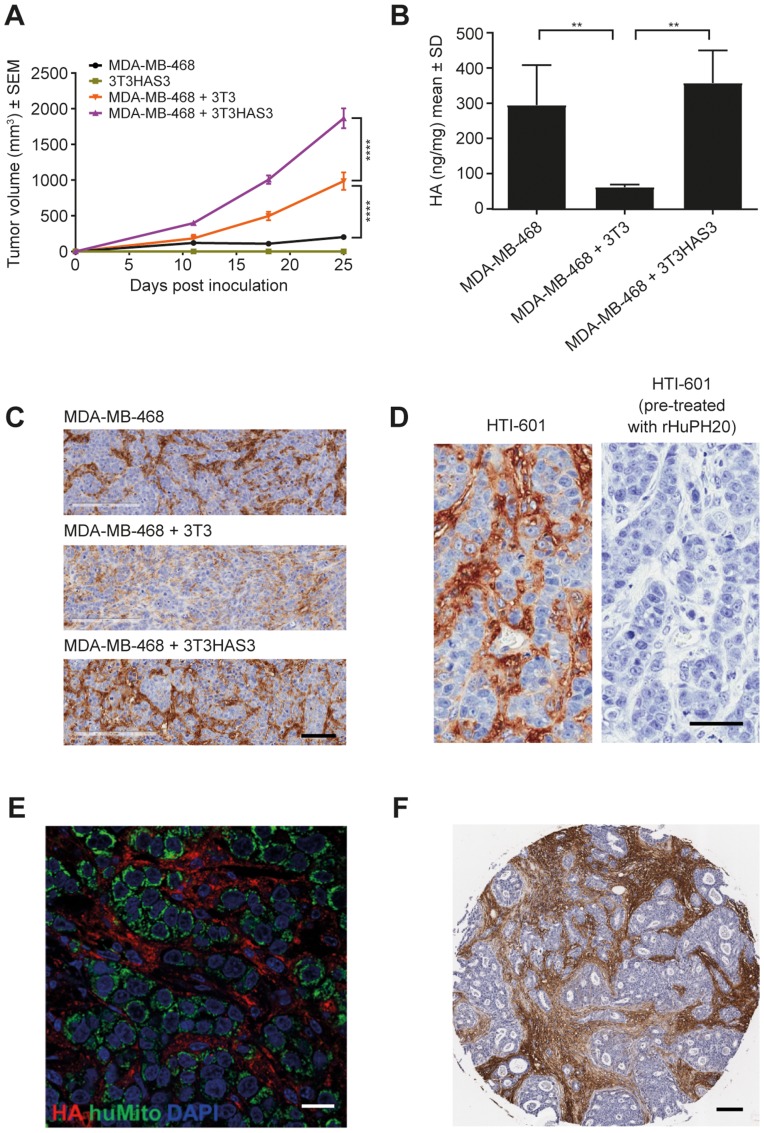
Engineered HA-accumulating fibroblast cells promoted *in vivo* tumor growth in the MDA-MB-468 breast cancer model. (**A**) The growth of MDA-MB-468 xenograft tumors was promoted by the presence of exogenous 3T3 fibroblast cells and further enhanced by the engineered HA-accumulating 3T3HAS3 cells, F(3, 26) = 106.1, *p* < 0.0001 for group effect (two-way ANOVA repeated measures). Group size *n* = 7–8. (**B**) MDA-MB-468 co-graft tumors with 3T3HAS3 synthesized higher levels of HA than co-graft tumors with 3T3 fibroblasts, F(2, 9) = 13.6, *p* = 0.0019 (one-way ANOVA). Tumors with MDA-MB-468 alone had similar levels of HA as MDA-MB-468 + 3T3HAS3 co-graft tumors, but it was not sufficient to drive tumor growth. (**C**) IHC HA staining of co-graft tumor samples with HTI-601 showed the presence of HA in the tumor stroma of MDA-MB-468 and MDA-MB-468 + 3T3HAS3 tumors. (**D**) No IHC signal was detected by HTI-601 when tumor sections (MDA-MB-468 + 3T3HAS3 shown as an example) were pre-treated with rHuPH20, demonstrating the specificity of HTI-601 toward HA. (**E**) Immunofluorescent staining of MDA-MB-468 + 3T3HAS3 co-graft tumors showed that tumor cells (green, identified by antibody against human mitochondria, HuMito) are surrounded by HA in the stroma (red, identified by HTI-601). Blue: DAPI. (**F**) Example of human breast invasive ductal carcinoma specimen (Grade 2, estrogen receptor-positive, and HER2-negative) with HA-accumulating stroma. Scale bars: 100 µm (C), 50 µm (D), 20 µm (E), and 100 µm (F). ^**^ *p* < 0.01 (Tukey’s post-test); ^****^ *p* < 0.0001 (Tukey’s post-test). Abbreviations: ANOVA, analysis of variance; DAPI, 4′,6-diamidino-2-phenylindole; HA, hyaluronan; HER-2, human epidermal growth factor receptor 2; IHC, immunohistochemistry; rHuPH20, recombinant human hyaluronidase PH20; SD, standard deviation; SEM, standard error of the mean; sHA, Select HA.

The triple-negative breast cancer model MDA-MB-468 was selected for this study for the following reasons: (1) MDA-MB-468 cells synthesize low levels of HA (Figure 1A); (2) MDA-MB-468 cells express CD44 [38], making them potential recipients of HA synthesized by stromal cells; and (3) MDA-MB-468 tumors do not grow well *in vivo* as a xenograft and potentiation by stromal HA could be easily measured.

An HA-accumulating stromal fibroblast cell model was engineered by transducing Balb/c 3T3 fibroblasts with a lentivirus encoding human HAS3 [37]. MDA-MB-468 and 3T3 cells synthesized low amounts of HA in the culture supernatant, whereas 3T3HAS3 cells synthesized abundant levels of HA in both the culture supernatant and the cell pellet (Figure 1A and 1B). HA synthesized by 3T3 cells and 3T3HAS3 cells in culture supernatant was of HMW (Figure 1C). Spiking of Select-HA™ 1000 kDa showed that ethanol precipitation and reconstitution of HA from culture supernatant did not affect the apparent size and recovery of HA (Figure 1C). In addition, the HA signal detected by this method was subject to degradation by hyaluronidase (Figure 1D).

To examine whether HA synthesized by the engineered 3T3HAS3 cells bound to MDA-MB-468 cells, 3T3HAS3 cells and MDA-MB-468 cells were co-cultured in a chamber slide. HA and MDA-MB-468 cells were then identified by immunofluorescent staining with DyLight 594-conjugated TSG-6-ΔHep-Fc (red) and anti-human mitochondria antibody (green), respectively (Figure 1E and 1F). Very little HA was detected when MDA-MB-468 cells (huMito+) were cultured alone (Figure 1E). However, in the presence of 3T3HAS3 fibroblasts (mouse origin, huMito− cells), pericellular HA signal was detected around MDA-MB-468 cells, suggesting that HA synthesized by 3T3HAS3 cells bound to MDA-MB-468 tumor cells (Figure 1F).

### Engineered 3T3HAS3 fibroblast cells promote the *in vivo* growth of the MDA-MB-468 model

To examine whether the engineered 3T3HAS3 cells supported the growth of MDA-MB-468 *in vivo*, 3T3HAS3 cells were co-grafted with MDA-MB-468 cells in the right hind leg adjacent to the tibial periosteum [3]. The peritibial tumor model has been shown to mimic the increased interstitial pressure that is associated with increased levels of HA in the TME [1, 3]. When inoculated alone, 3T3HAS3 cells did not form detectable tumors, and xenograft tumors with only MDA-MB-468 cells grew very slowly (Figure 2A). In the presence of the parental 3T3 cells, MDA-MB-468 xenograft tumors grew much faster, and tumor growth was further enhanced when 3T3HAS3 cells were co-grafted (Figure 2A). HA levels in MDA-MB-468 + 3T3HAS3 co-graft tumors were higher than that in MDA-MB-468 + 3T3 tumors (Figure 2B). MDA-MB-468 tumors contained a similar concentration of HA as MDA-MB-468 + 3T3HAS3 co-grafts at the end of the study (Figure 2B). IHC analysis showed that the HA signal was present in the tumor stroma, suggesting MDA-MB-468 tumors recruited HA-accumulating cells during *in vivo* growth (Figure 2C). As such, these data suggest that: (1) increasing HA levels alone was not sufficient to promote tumor growth; and (2) co-grafting of 3T3 cells generated a pro-tumor TME that can be further enhanced by HA accumulation through HAS3 overexpression.

IHC analysis with the HA-probe HTI-601 was used to assess the distribution of HA within MDA-MB-468 + 3T3HAS3 co-graft tumors [39]. HA was found to associate with the tumor stroma, surrounding clusters of tumor cells (Figure 2C and 2D). Immunofluorescent staining with a marker specific for human mitochondria confirmed the presence of human tumor cells in the xenograft (Figure 2E, green). Again, HA was found surrounding clusters of tumor cells (Figure 2E, red). This morphology was very similar to that described in certain human breast cancer specimens, with HA specifically associated with tumor stroma [26]. An example of human breast invasive ductal carcinoma (grade 2, estrogen receptor-positive, and HER2-negative) is shown in Figure 2F, in which the IHC signal for HA was mostly associated with tumor stroma.

### Degradation of HA by PEGPH20 or co-expression of PH20-FL in 3T3HAS3 cells inhibited the growth of the co-graft model

**Figure 3:**
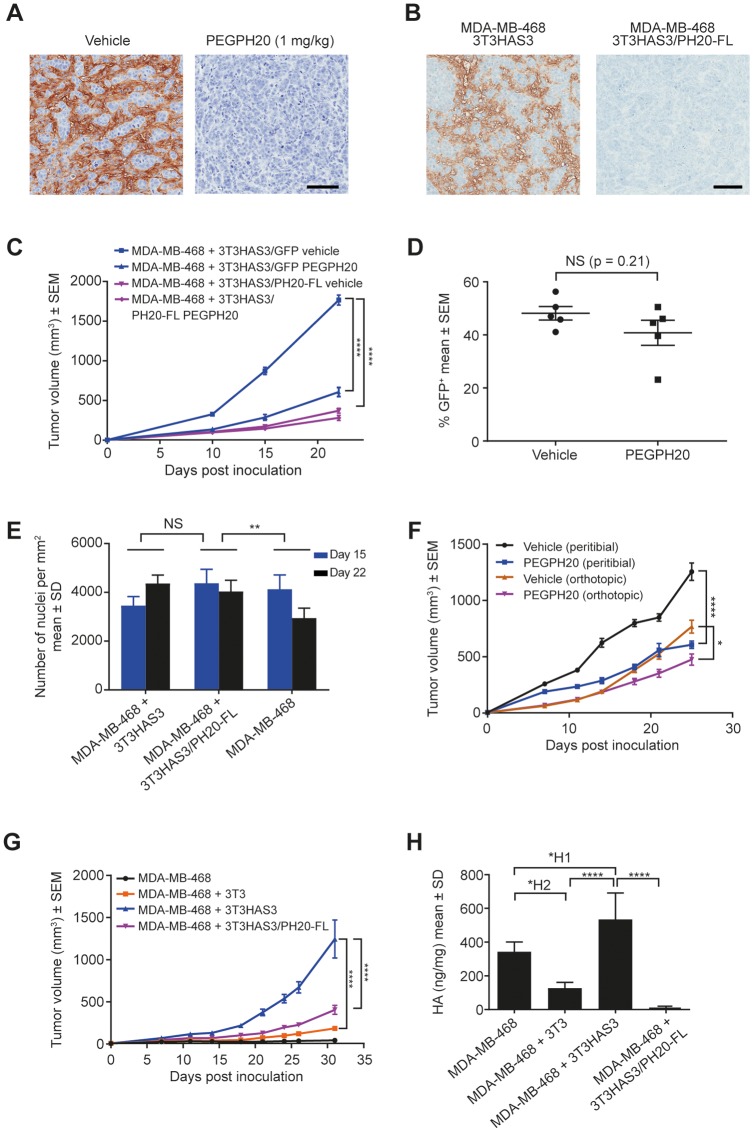
Tumor growth in the breast cancer co-graft model with HA-accumulating stroma was dependent on HA synthesized by the engineered stromal fibroblast cells. (**A**) Enzymatic degradation of HA in MDA-MB-468 + 3T3HAS3 co-graft tumors by PEGPH20 at 1 mg/kg. (**B**) Enzymatic degradation of HA by expression of PH20-FL protein in 3T3HAS3 cells. (**C**) Tumor growth of the MDA-MB-468 + 3T3HAS3 co-graft peritibial model was significantly inhibited in response to twice weekly dosing of PEGPH20 (1 mg/kg) starting on the day prior to cell inoculation, or by the expression of the full-length *PH20* gene (*PH20-FL*) in 3T3HAS3 cells, F(3, 20) = 165.8, *p* < 0.0001 (two-way ANOVA repeated measures). Group size *n* = 6. (**D**) Quantification of 3T3HAS3/GFP cells by flow cytometry showed that the contribution of engineered stromal fibroblasts to the total CD45 negative (CD45-) cell population did not change in response to PEGPH20 treatment (*p* = 0.21). (**E**) Similar cell density in MDA-MB-468 + 3T3HAS3 and MDA-MB-468 + 3T3HAS3/PH20-FL co-graft tumors. Cell density of MDA-MB-468 tumors was lower than that of MDA-MB-468 + 3T3HAS3/PH20-FL tumors, F(2, 30) = 6.159, *p* = 0.0057 (two-way ANOVA). (**F**) Growth of co-graft tumors was inhibited by PEGPH20 treatment with both peritibial inoculation (F(1, 14) = 68.79, *p* < 0.0001) and orthotopic mammary fat pad inoculation (F(1, 14) = 8.722, *p* = 0.0105). Group size *n* = 8. (**G**) Tumor growth of the MDA-MB-468 + 3T3HAS3 orthotopic co-graft model was significantly inhibited when 3T3HAS3 cells were engineered to express PH20-FL, F(3, 28) = 61.72, *p* < 0.0001 (two-way ANOVA repeated measures). Group size *n* = 8. (**H**) Co-graft tumors with 3T3HAS3 contained higher levels of HA than co-graft tumors with 3T3 or 3T3HAS3/PH20-FL fibroblast cells, F(3, 20) = 39.18, *p* < 0.0001 (one-way ANOVA). Scale bars: 100 μm (A & B). NS, not significant; ^*^ *p* = 0.0105; ^**^ *p* < 0.01; ^****^ *p* < 0.0001 (Tukey’s post-test); ^*H1^ *p* = 0.0199; ^*H2^ *p* = 0.0125. Abbreviations: ANOVA, analysis of variance; HA, hyaluronan; PEGPH20, pegvorhyaluronidase alfa; SD, standard deviation; SEM, standard error of the mean.

To assess the functional role of HA synthesized by the engineered 3T3HAS3 cells, 2 different approaches were used to degrade HA. First, PEGPH20 was administered to mice bearing MDA-MB-468 + 3T3HAS3 co-graft tumors. All mice received either the vehicle control or PEGPH20 at 1 mg/kg twice weekly, starting 1 day prior to cell inoculation to ensure the presence of PEGPH20 at the time of inoculation and throughout the study duration. Second, 3T3HAS3 cells were engineered to express the full length SPAM1/PH20 (PH20-FL) protein. Both approaches led to efficient HA degradation in tumor samples (Figure 3A, PEGPH20 treatment; Figure 3B, 3T3HAS3/PH20-FL).

PEGPH20 treatment significantly delayed xenograft tumor growth (Figure 3C, comparing MDA-MB-468 + 3T3HAS3/GFP vehicle vs. PEGPH20). Similarly, when 3T3HAS3 cells were engineered to express PH20-FL, its effect on promoting the growth of the co-graft model was diminished (Figure 3C, comparing MDA-MB-468 + 3T3HAS3/GFP vehicle vs. MDA-MB-468 + 3T3HAS3/PH20-FL vehicle). Flow cytometry analysis of xenograft tumors showed that the percentage of 3T3HAS3/GFP cells did not change significantly with chronic PEGPH20 dosing (Figure 3D, *p* = 0.21, unpaired two-tailed *t*-test). To investigate whether changes in tumor size were associated with a difference in tumor burden, tumor cell nuclear density was analyzed from xenograft tumor samples obtained from an independent study. MDA-MB-468 + 3T3HAS3 and MDA-MB-468 + 3T3HAS3/PH20-FL co-graft tumors displayed similar cell density (Figure 3E). These data support the hypothesis that the larger volume of MDA-MB-468 + 3T3HAS3 co-graft tumors was likely associated with a greater number of tumor cells.

PEGPH20 administration inhibited the growth of the co-graft tumor when MDA-MB-468 + 3T3HAS3 cells were inoculated either peritibially or in the mammary fat pad (Figure 3F). Likewise, 3T3HAS3/PH20-FL was much less efficient in promoting the growth of the co-graft in the mammary fat pad, compared with 3T3HAS3 (Figure 3G). HA quantification of the xenograft tumors confirmed the degradation of HA in MDA-MB-468 + 3T3HAS3/PH20-FL co-grafts (Figure 3H). These data demonstrate that HA synthesized by the engineered 3T3HAS3 cells plays a critical role in enhancing the growth of the MDA-MB-468 tumor cells *in vivo*.

### Knocking down CD44 expression in MDA-MB-468 cells did not affect xenograft tumor growth

**Figure 4:**
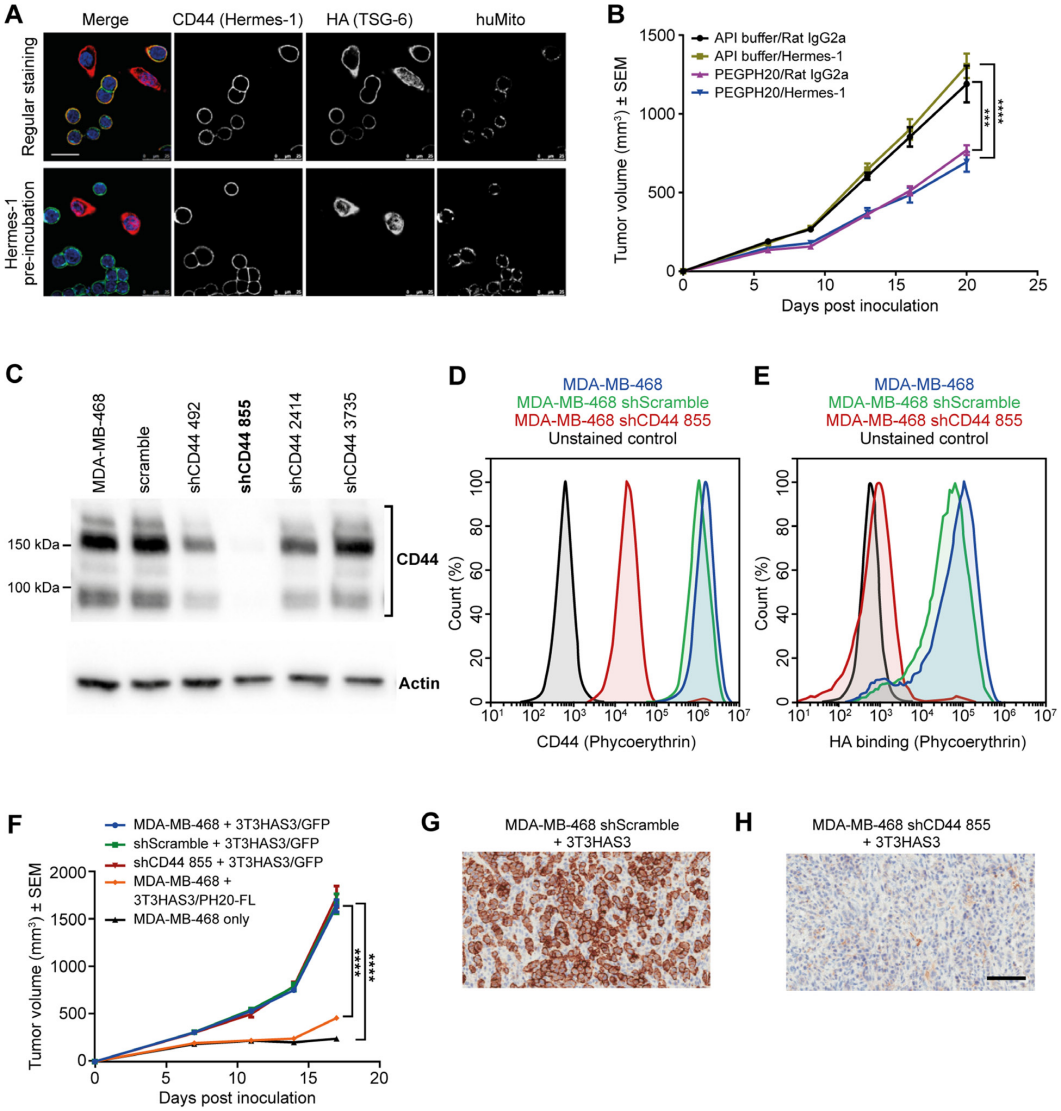
Tumor growth in the breast cancer co-graft model with HA-accumulating stroma was independent of CD44 expression in MDA-MB-468 tumor cells. (**A**) Anti-CD44 antibody Hermes-1 specifically bound to MDA-MB-468 cells (upper panel) and blocked the binding of HA to MDA-MB-468 cells (lower panel). Blue: DAPI, green: human CD44 (Hermes-1 antibody), red: HA (TSG-6), and teal: human mitochondria. (**B**) The growth of MDA-MB-468 + 3T3HAS3 tumors was inhibited by PEGPH20 but not by anti-CD44 treatment. Anti-CD44 antibody (Hermes-1, 30 mg/kg) was given on a twice-weekly schedule starting 1 day prior to inoculation, with or without concomitant dosing of PEGPH20 (0.0375 mg/kg), F(3, 28) = 19.67, *p* < 0.0001 (two-way ANOVA repeated measures). Group size *n* = 8. (**C**) Lentiviral shRNA vectors were generated to knockdown the expression of CD44 in MDA-MB-468 cells. Only 1 out of the 4 vectors tested had a nearly complete knockdown of CD44 level (shCD44 855). (**D**) Flow analysis confirmed diminished CD44 expression in MDA-MB-468 sh855 cells (blue: MDA-MB-468, green: MDA-MB-468 shScramble, red: MDA-MB-468 shCD44 855, and black: unstained control of MDA-MB-468). (**E**) MDA-MB-468 shCD44 855 cells showed diminished binding to HA (blue: MDA-MB-468, green: MDA-MB-468 shScramble, red: MDA-MB-468 shCD44 855, and black: unstained control of MDA-MB-468). (**F**) Knockdown of CD44 expression in MDA-MB-468 cells did not affect the growth of the co-graft, whereas expression of PH20-FL in 3T3HAS3 significantly inhibited tumor progression (MDA-MB-468 + 3T3HAS3 vs. MDA-MB-468 + 3T3HAS3/PH20-FL), F(4, 34) = 125.7, *p* < 0.0001 (two-way ANOVA repeated measures). Group size *n* = 7–8. (**G–H**) IHC of xenograft samples confirmed the presence of CD44 in shScramble (G) and loss of CD44 in shCD44 855 (H) tumor samples. Scale bars: 25 µm (A), 100 µm (G, H). ^***^ *p* = 0.0002; ^****^ *p* < 0.0001 (Tukey’s post-test). Abbreviations: DAPI, 4′,6-diamidino-2-phenylindole; PEGPH20, pegvorhyaluronidase alfa; sh, small hairpin.

As discussed earlier, MDA-MB-468 cells express CD44. Immunofluorescent staining of the co-culture of MDA-MB-468 and 3T3HAS3 cells showed co-localization of HA and CD44 on the membrane of MDA-MB-468 cells (Figure 4A, regular staining). When the co-culture was incubated with the anti-human CD44 antibody Hermes-1 for 1 hour prior to cell fixation, the co-localization of HA with CD44 was no longer observed (Figure 4A, Hermes-1 pre-incubation), demonstrating the requirement for CD44 to mediate an association between HA and MDA-MB-468 cells in culture.

Next, the anti-human CD44 antibody Hermes-1 was used to evaluate the dependence of the MDA-MB-468 + 3T3HAS3 co-graft model on the HA-CD44 interaction. When dosed at 30 mg/kg, the Hermes-1 antibody did not show an independent effect on the growth of the MDA-MB-468 + 3T3HAS3 co-graft, even when mice were dosed in combination with PEGPH20 in order to decrease HA levels in the xenograft tumors (Figure 4B). In comparison, tumor growth was reduced in mice treated with PEGPH20 (0.0375 mg/kg, a dose level equivalent to the dose level being studied in human patients).

To assess whether CD44 expression in MDA-MB-468 cells is required for the growth of the co-graft tumor, CD44 expression was knocked down in MDA-MB-468 cells with lentiviral transduction of small hairpin RNA (shRNA) against CD44. Of the 4 shCD44 vectors tested, only 1 resulted in near complete loss of CD44 (Figure 4C, shCD44 855). Significant decrease of CD44 expression with shCD44 855 was further confirmed by flow cytometry (Figure 4D). In addition, MDA-MB-468 shCD44 cells showed diminished binding to HA (Figure 4E). Knockdown of CD44 expression in MDA-MB-468 cells did not affect tumor growth *in vivo* (Figure 4F). In contrast, MDA-MB-468 + 3T3HAS3/PH20-FL co-graft tumors grew much slower than MDA-MB-468 +3T3HAS3 tumors (Figure 4F), consistent with the effects of the hyaluronidase as shown in Figure 3C and 3D. The average tumor size of MDA-MB-468 + 3T3HAS3/PH20-FL co-graft tumors was only 28% of that of MDA-MB-468 + 3T3HAS3 co-graft tumors on day 18. IHC of xenograft tumor samples confirmed that human CD44 protein was nearly undetectable in co-grafts with MDA-MB-468 shCD44 855 (Figure 4G and 4H, shScramble vs. shCD44 855). Together, these data suggest that neither CD44 expression by MDA-MB-468 tumor cells, nor its interaction with HA, is necessary for the enhanced tumor growth in the presence of the engineered 3T3HAS3 fibroblast cells.

### Loss of CD44 expression in 3T3HAS3 fibroblasts had minimal impact on the growth of MDA-MB-468 co-graft tumors

**Figure 5:**
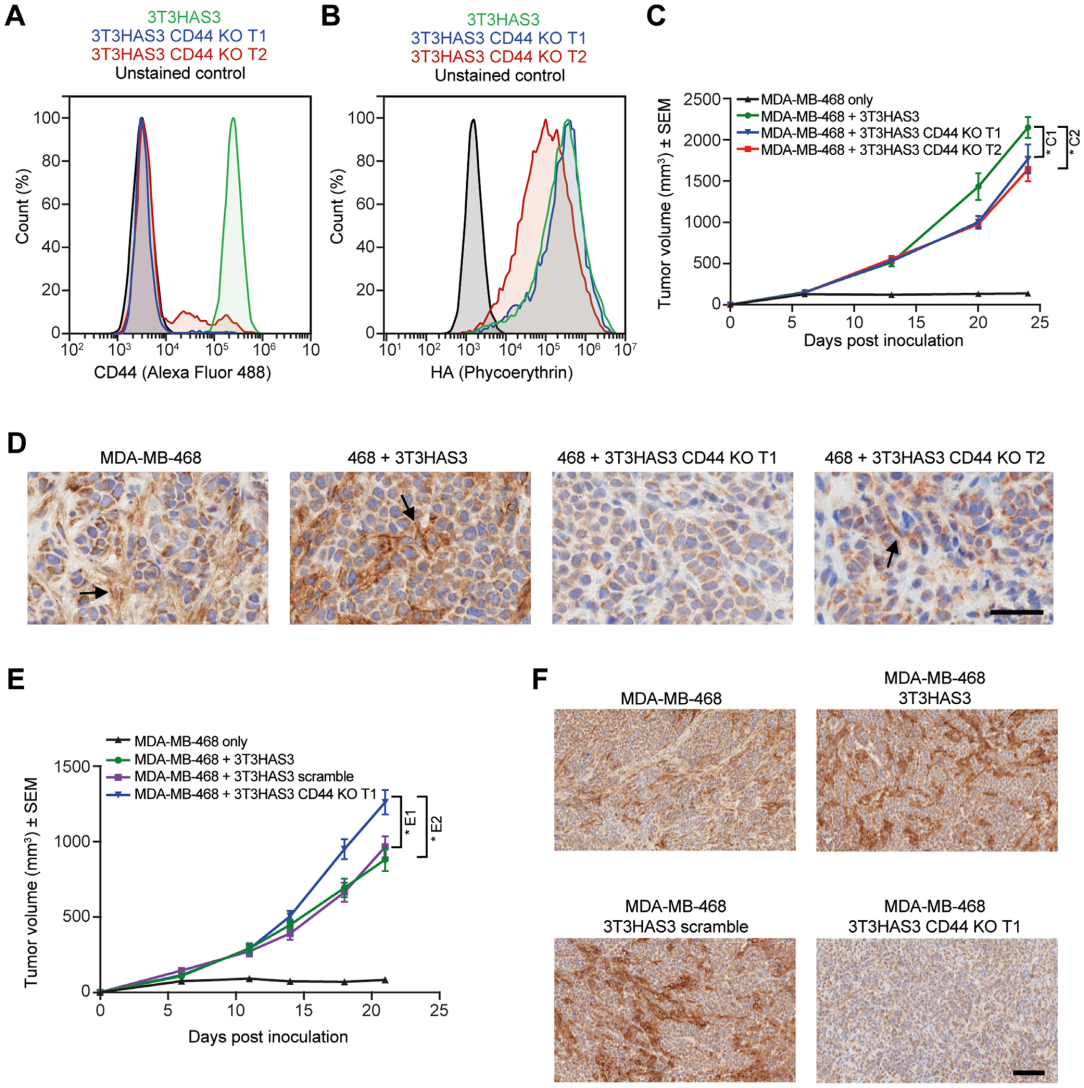
Tumor growth in the breast cancer co-graft model with HA-accumulating stroma was independent of CD44 expression in 3T3HAS3 fibroblast cells. (**A**) Flow cytometry analysis of CRISPR-mediated 3T3HAS3 CD44 knockout cells (green: 3T3HAS3, blue: 3T3HAS3 CD44 KO T1, red: 3T3HAS3 CD44 KO T2, black: unstained control of 3T3HAS3) confirmed loss of CD44 expression, with 96% and 78% of the cell population negative for 3T3HAS3 CD44 KO T1 and T2 cells, respectively. (**B**) HA level associated with 3T3HAS3 was not changed upon loss of CD44, especially in CD44 KO T1 cells (3T3HAS3 CD44 KO T1 vs. 3T3HAS3). (**C**) Tumor growth of MDA-MB-468 co-grafts with 3T3HAS3 CD44 KO cells was slightly reduced compared with 3T3HAS3 control co-graft tumors, F(3, 28) = 62.12, *p* < 0.0001 (two-way ANOVA repeated measures). Group size *n* = 8. (**D**) MDA-MB-468 co-grafts with 3T3HAS3 CD44 KO T1 did not have a detectable CD44 signal and those with 3T3HAS3 CD44 T2 had residual but infrequent CD44 signal in the tumor stroma. The arrows indicate the CD44 IHC signal from tumor stroma. 3T3HAS3 appeared to have a stronger CD44 expression than endogenous fibroblast cells recruited by MDA-MB-468 cells. (**E**) In an independent study, tumor growth of MDA-MB-468 co-grafts with 3T3HAS3 CD44 KO T1 was slightly increased compared with MDA-MB-468 co-grafts with 3T3HAS3 or 3T3HAS3 scramble controls, F(3, 28) = 48.20, *p* < 0.0001 (two-way ANOVA repeated measures). Group size *n* = 8. (**F**) MDA-MB-468 co-graft with 3T3HAS3 CD44 KO T1 did not show a detectable CD44 signal in the tumor stroma, in contrast to co-graft tumors with 3T3HAS3 or 3T3HAS3 scramble. Scale bars: 50 μm (D) and 100 μm (F). ^*C1^ *p* = 0.0482; ^*C2^ *p* = 0.018 (Tukey’s post-test); ^*E1^ *p* = 0.0392; ^*E2^ *p* = 0.0350 (Tukey’s post-test). Abbreviations: ANOVA, analysis of variance; HA, hyaluronan; IHC, immunohistochemistry.

To determine whether the role of stromal HA is mediated via “autocrine” signaling through interactions between HA and CD44 on the stromal cells, we used a clustered regularly interspaced short palindromic repeat (CRISPR)-based lentiviral vector to knock down CD44 expression in 3T3HAS3 cells. Of the 3 different target sequences tested, Targets 1 and 2 (T1 and T2) yielded ~96% and ~78% CD44-negative 3T3HAS3 cells, respectively. This is shown by the flow cytometry analysis, carried out with a CD44 antibody (IM7 clone) that recognizes both human and mouse CD44 proteins (Figure 5A and 5B, blue: T1, red: T2). When 3T3HAS3 CD44 CRISPR KO (3T3HAS3 CD44 KO) cells and MDA-MB-468 cells were used together to inoculate mice, both T1 and T2 3T3HAS3 CD44 KO cells were able to significantly promote the growth of MDA-MB-468 tumors (Figure 5C, MDA-MB-468 vs. the rest of study groups). As shown in Figure 5C, the tumor growth of MDA-MB-468 co-grafts with 3T3HAS3 CD44 KO T1 and T2 was slightly reduced when compared with MDA-MB-468 + 3T3HAS3 co-grafts (at last data point analyzed, the average tumor sizes of T1 and T2 co-grafts were 82.2% and 76.4% of MDA-MB-468 + 3T3HAS3 co-grafts, respectively). Significant decrease (T2) or near loss (T1) of CD44 signal in the tumor stroma was confirmed by IHC with antibodies specific for mouse CD44 protein (Figure 5D). In a separate experiment, tumor growth of MDA-MB-468 + 3T3HAS3 CD44 KO T1 co-grafts was slightly greater than that of MDA-MB-468 + 3T3HAS3 co-graft tumors (Figure 5E, *p* < 0.05). The loss of CD44 expression in MDA-MB-468 + 3T3HAS3 CD44 KO T1 was confirmed by CD44 staining (Figure 5F). Taken together, these data demonstrated that loss of CD44 expression in the engineered stromal fibroblast 3T3HAS3 cells did not have a major impact on the growth of MDA-MB-468 + 3T3HAS3 co-graft tumors.

### Loss of CD44 expression in the host did not inhibit the growth of MDA-MB-468 co-graft tumors

**Figure 6:**
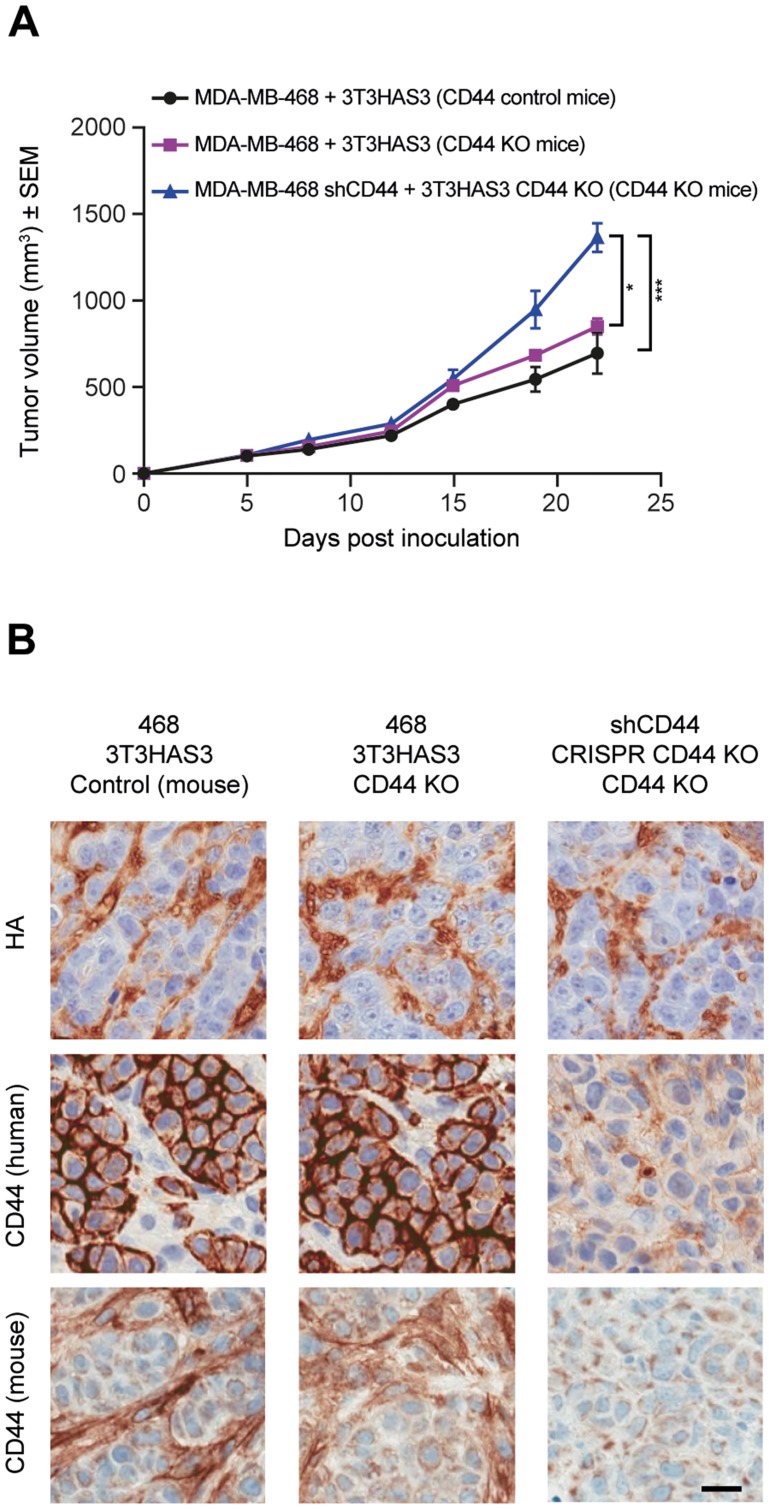
Tumor growth in the breast cancer co-graft model with HA-accumulating stroma was independent of CD44 expression in MDA-MB-468 tumor cells, 3T3HAS3 fibroblast cells, and in the host. (**A**) Tumor growth curve of MDA-MB-468 + 3T3HAS3 co-grafts in CD44 control and KO nude mice, and of MDA-MB-468 shCD44 + 3T3HAS3 CD44 KO in CD44 KO mice, F(2, 13) = 12.45, *p* = 0.001 (two-way ANOVA repeated measures). Group size *n* = 5–6. (**B**) Immunohistochemistry analysis of tumor samples confirmed the presence of HA in all 3 tumors and loss of human and mouse CD44 expression in tumor samples derived from MDA-MB-468 shCD44 + 3T3HAS3 CD44 KO in CD44 nude mice. Scale bars (B): 25 μm. ^*^ *p* = 0.0189; ^***^ *p* = 0.0007 (Tukey’s post-test). Abbreviations: HA, hyaluronan; SEM, standard error of the mean; sh, small hairpin.

To determine whether expression of CD44 in the host is needed for the growth of the MDA-MB-468 co-graft tumors, CD44 KO nude mice were generated by crossing CD44 KO mice to NCr nu/nu mice for two generations. CD44 KO nude mice from F3/F4 inbreeding were used for this study, with both wild-type and heterozygous littermates as controls. The MDA-MB-468 + 3T3HAS3 co-graft tumors grew similarly in CD44 KO nude mice and in control mice (Figure 6A). Furthermore, when MDA-MB-468 shCD44 + 3T3HAS3 CD44 KO T1 tumors were co-grafted into CD44 KO nude mice, tumor growth was increased when CD44 expression was lost or diminished (Figure 6A). Loss of CD44 expression was confirmed by IHC analysis; neither human nor mouse CD44 was detected in MDA-MB-468 shCD44 + 3T3HAS3 CD44 KO T1 co-graft tumors from the CD44 KO nude mice (Figure 6B). In comparison, no obvious differences in HA levels were observed in these xenograft tumors (Figure 6B, top panels). Of note, mouse CD44 was detected in the stroma of MDA-MB-468 + 3T3HAS3 co-graft tumors in the CD44 KO nude mice, suggesting that 3T3HAS3 cells were contributing to the stromal content in the co-graft tumors.

## Discussion

We described a novel model to understand the role of HA accumulation in the TME. In this model, 3T3 fibroblast cells were engineered to express HAS3 and accumulate HA in the TME. The 3T3HAS3 fibroblasts produced HMW HA in cell culture and promoted tumor growth when co-grafted with human triple-negative breast cancer cells MDA-MB-468 in nude mice. The pro-tumor effect of the HA-accumulating fibroblasts was highly dependent on HA, as enzymatic degradation of HA by either co-expression of the full-length human *SPAM1* gene (PH20-FL) or by systemic administration of PEGPH20 significantly hindered tumor growth. Unexpectedly, this effect did not appear to require CD44 expression, the well-characterized receptor for HA. This was demonstrated by knocking down CD44 expression in the MDA-MB-468 cancer cells, by knocking out CD44 in the 3T3HAS3 fibroblast cells, and by grafting these cells into the nude mice host system.

CD44 is considered a cancer stem cell marker in many tumor types and has been shown to signal through multiple pro-cancer pathways, including Ras/MAPK, PI3K/Akt, and Rho GTPases. Furthermore, CD44 expression is suppressed by tumor suppressor p53 [40]. Unexpectedly, targeting HA-CD44 interaction, or even the expression of CD44 itself, did not measurably interfere with the growth of the xenograft tumors. However, the lack of an effect from the loss of CD44 on primary tumor growth has been previously described in genetically-modified mouse models [41–43]. In addition, although a body of literature supports a role for CD44 in metastasis, there are also data describing an inhibitory effect of CD44 on tumor metastasis [41, 43, 44]. Indeed, the prognostic value of CD44 expression is unclear [45, 46]. These seemingly conflicting reports may be a result of the intrinsic complexity of the CD44 molecule.

Although highly conserved, CD44 is subject to post-transcriptional regulation and post-translational modification. There are as many as 20 different splice variants of CD44, which can be further modified by glycosylation and chondroitin sulfate binding [45]. The standard and variant forms of CD44 were reported to have opposite functions in tumorigenesis and may respond differently to CD44 blockade [47, 48]. MDA-MB-468 cells were reported to express predominantly CD44v isoforms [48]. Our experimental data showed that at least 4 bands were clearly identifiable from the anti-CD44 western blot (Figure 4C). Knocking down the expression of all CD44 isoforms might result in the cancellation of positive and negative effects. To confound the complexity of CD44 itself, tumor cells often carry mutations that bypass signaling at the receptor level. For example, MDA-MB-468 cells have a 44-bp deletion in the phosphatase and *tensin homolog (PTEN)* gene and display elevated phosphorylation and activation of Akt [49, 50]. As a result, these cells may be less sensitive to signal ablation at the cell surface. Since pathways such as Ras and PI3K/Akt are often dysregulated in tumor models, blocking CD44 alone may not be effective in inhibiting these amplified pro-tumorigenesis signals. In addition, the 3T3 cells used in this experimental system may provide high levels of certain growth factors in the TME. CD44 is known to be a co-receptor for a number of receptor tyrosine kinases, such as c-MET, vascular endothelial growth factor receptor (VEGFR), platelet-derived growth factor receptor beta (PDGFR-ß), fibroblast growth factor receptor (FGFR), and epidermal growth factor receptor (EGFR), and is suggested to integrate extracellular matrix cues [51] and possibly lower the activation threshold of growth factor receptors. This function may be more important when the level of a growth factor is a limiting factor in the experimental system, but not easily discernible in a TME in which the level of growth factors exceed the activation threshold of their receptors.

Despite no detectable effect from knocking down/out CD44 expression in this experimental system, enzymatic degradation of HA consistently inhibited the growth of the engineered xenograft tumor model with HA-accumulating stroma. This was demonstrated by either the co-expression of hyaluronidase (PH20-FL) in 3T3HAS3 cells or by systemic administration of PEGPH20. Notably, although MDA-MB-468 xenograft tumors without 3T3HAS3 accumulated HA, their growth was much slower than that of MDA-MB-468 + 3T3HAS3 co-grafts. In comparison, MDA-MB-468 + 3T3 tumors did not accumulate as much HA and grew faster than MDA-MB-468-only tumors. As discussed above, in addition to HA, other factors may be provided by 3T3HAS3 cells to assist tumor growth, and HA may work in combination with these additional factors to provide an optimal TME for the growth of MDA-MB-468 tumors. For example, HAS3 expression in colon cancer cell line SW620 was required for matrix retention [52]. PEGPH20-mediated HA degradation in mouse syngeneic CT26/HAS3 tumors was associated with a decrease in VEGF-A165 levels in the TME [53]. It is possible that 3T3HAS3 cells express certain growth factors that might be retained more efficiently in an HA-rich TME.

An alternative explanation to the observation that the level of HA in MDA-MB-468 + 3T3 co-graft tumors was lower than that of MDA-MB-468 tumors may be that 3T3 cells might express hyaluronidases such as Hyal 1. Hyal 1 was shown to promote tumor growth when expressed at moderate levels and inhibit tumor growth when expressed at high levels [54]. In the MDA-MB-468 + 3T3HAS3 experimental system, expression of the PH20 hyaluronidase in 3T3HAS3 fibroblasts abolished its pro-tumor activity (Figures 3C, 3G, and 4F). Furthermore, administration of PEGPH20 inhibited the growth of co-graft tumors at 0.0375 mg/kg (Figure 4B) and 1 mg/kg (Figure 3C and 3F) dose levels.

In addition to receptor signaling, HA synthesis is intricately associated with cellular metabolism. The process of HA polymerization uses UDP-glucuronic acid (UDP-GlcUA) and UDP-*N*-acetylglucosamine (UDP-GlcNAc) as substrate building blocks, both of which are derived from glycolysis intermediates [55]. Interestingly, HA degradation was shown to have a general effect on glycolysis in tumor and non-tumor cells *in vitro* [56]. Although it is not fully understood how HA degradation could affect cellular metabolism, it is reasonable to hypothesize that HA degradation by a high dose of PEGPH20 may shift the balance of HA catabolism and cellular metabolism. Further exploration of the interaction between HA degradation and cellular metabolism as well as signaling through other HA receptors, such as HA-mediated motility receptor (RHAMM), may provide more insight into the understanding of a pro-tumor TME with an HA-rich stroma and how such a TME can be more effectively targeted by anti-cancer therapies.

In conclusion, our results demonstrate that the tumor growth of an engineered xenograft breast cancer model with an HA-accumulating stroma is dependent on HA and independent of CD44, suggesting that HA-CD44 interaction may not be the main mechanism through which HA promotes tumor growth in certain tumors.

## Materials and methods

### Cell culture

MDA-MB-468 and Balb/c 3T3 cells were obtained from ATCC^®^ (Manassas, VA, USA). MDA-MB-468 and 3T3HAS3 cells were authenticated based on short tandem repeat profiles (IDEXX, Columbia, MO, USA) and were negative for mycoplasma (MycoAlert, Lonza, Allendale, NJ, USA). MDA-MB-468 cells were maintained in Roswell Park Memorial Institute (RPMI) medium supplemented with 10% fetal bovine serum, and 3T3 lines were maintained in Dulbecco’s Modified Eagle Medium (DMEM) supplemented with 5% fetal calf serum. Co-cultures of MDA-MB-468 and 3T3HAS3 cells were maintained in RPMI supplemented with 10% fetal bovine serum.

### Cell line engineering

Cell lines were engineered through standard lentiviral transduction protocol. Replication incompetent lentiviruses encoding the *HAS3* gene, and full-length *sperm adhesion molecule 1* gene (*SPAM1*, which encodes the protein hyaluronidase PH20; PH20-FL) or green fluorescent protein (GFP), were generated with pLV-EF1a-IRES-Hyg and pLV-EF1a-IRES-Puro vectors, respectively (Biosettia, San Diego, CA, USA). A total of 4 shRNA vectors were designed to knock down the expression of human CD44 using the pLV-mU6-EF1a-GFP-puro vector (Biosettia, San Diego, CA, USA). MDA-MB-468 cells transduced with shCD44 855 (target sequence GGACCAATTACCATAACTA) were selected based on western blot analysis of CD44 expression (rabbit polyclonal antibody, Abcam, Cambridge, MA, USA). Lentiviral CRISPR vectors (pLenti-U6-sgRNA-SFFV-Cas9-2A-Puro) of mouse CD44 were obtained from Abmgood (British Columbia, Canada) and the target sequences were 95-GAATACACCTGCGTAGCGGC (Target 1) and 315-CGAGGATATATACTCCTGTG (Target 2).

### HA quantification

Hyaluronan DuoSet (R&D systems, Minneapolis, MN, USA) was used to quantify HA levels in culture samples and xenograft tumor samples. For quantification of HA in culture samples, cells (5 × 10^5^ in total) were plated in T75 culture flasks in a total volume of 20 ml and allowed to expand over 72 hours. Culture supernatant was then collected and filtered (0.22 µm filters) to remove cell debris. The cell pellet was digested with proteinase K (1 mg/ml proteinase K in 50 mM Tris, 20 mM CaCl_2_, and 2 mM MgCl_2_) at 55°C for 16 to 24 hours. For quantification of HA levels in xenograft tumor samples, tumor specimens were collected by snap freezing in liquid nitrogen and digested with proteinase K working solution (40 µl of proteinase K working solution/mg tumor tissue) at 55°C for 24 hours. Processed samples were then incubated for 20 minutes at 90 to 100°C to inactivate proteinase K and centrifuged at 10,000 rpm for 20 min (4 to 15°C) to remove debris. HA levels were expressed as µg/1 × 10^6^ cells for cell culture samples and ng/mg for tumor samples.

### HA size determination of culture supernatant

Conditioned media was treated with proteinase K, and HA was then precipitated with equal volume of 100% ethanol and resuspended in deionized H_2_O. Processed samples were separated on 0.4% agarose gel (SeaKem Gold, Lonza, Allendale, NJ, USA) in 1x tris acetate EDTA buffer (100V for 75 minutes) with ice blocks to maintain gel temperature, followed by a Southern blot transfer process to HyBond+ membrane in 100 mM tris acetate, pH 7.3 for 16 hours. HA was visualized on the membrane by western blot analysis. Specifically, 2% milk in phosphate-buffered solution (PBS) 0.05% Tween 20 was used as blocking reagent and as reagent diluent. Instead of using a primary antibody, biotin-TSG6-ΔHep-Fc (39) (0.5 µg/ml) was used to bind HA molecules on the membrane and horseradish peroxidase (HRP)-conjugated streptavidin was used for detection. To demonstrate that the sample processing procedure did not affect HA size, Select-HA 1000K (Hyalose, Oklahoma City, OK, USA) was spiked into conditioned media prior to sample processing. The specificity of biotin-TSG6-ΔHep-Fc was demonstrated by an HA blot of conditioned media that was pre-treated with hyaluronidase, *Streptomyces hyaluronlyticus* nov. species (1 unit/300 µl culture media, 37°C for 1 hour; EMD Millipore, Burlington, MA, USA).

### Tumor xenograft models

6- to 8-week-old female nude mice (NCr nu/nu, Taconic or athymic nude, Charles River Laboratories, Wilmington, MA, USA) were used for xenograft studies. Mice were handled in accordance with protocols detailed by the Institutional Animal Care and Use Committee. A mixture of MDA-MB-468 cells (or derivatives, 3 × 10^6^) and 3T3HAS3 cells (or derivatives, 2 × 10^6^ or 1 × 10^6^) were inoculated at a 0.05 ml final volume in the right hind leg adjacent to the tibia periosteum. Tumor volume was monitored by ultrasound imaging (VisualSonics, Ontario, Canada) for up to 4 weeks, or until tumor volume reached 1500 to 2000 mm^3^. For orthotopic inoculations, the same number of cells were mixed with an equal volume of ice cold matrigel and were inoculated into the fat pad of the fourth left nipple at a 0.1 ml final volume. Tumor growth was monitored by caliper measurement and tumor volume was estimated using the formula “½ × length × width^2^”. For studies with PEGPH20 treatment, mice were administered vehicle control (10 mM histidine, 130 mM NaCl, pH 6.5) or PEGPH20 (formulated in vehicle control at 1 mg/kg or 0.0375 mg/kg) 24 hours prior to cell inoculation. Hermes-1 antibody (30 mg/kg, BioXCell, West Lebanon, NH, USA) was used for *in vivo* and *in vitro* study to block HA-CD44 interaction. Mice were dosed intravenously on a twice weekly schedule that started 1 day prior to cell inoculation unless stated otherwise. The groups for the tumor growth studies consisted of 5 to 8 mice. CD44 knockout (KO) mice were obtained from the Jackson Laboratory (Bar Harbor, ME, USA). CD44 KO mice were crossed to NCr nu/nu mice (Taconic, Rensselaer, NY, USA) for 2 generations. CD44 KO nude mice from F3/F4 inbreeding were used for study, with both wild-type and heterozygous littermates as controls.

### Immunohistochemistry and immunofluorescent staining of xenograft tissue samples

Tumor tissue samples were fixed in 10% formalin. Sections of 5 µM thickness were processed as previously described [39]. Biotin-TSG-6-ΔHep-Fc (HTI-601) was used as primary probe for detection of HA [39]. Other antibodies used included anti-human mitochondria antibody (Abcam, Cambridge, MA, USA), anti-human CD44 antibody (Vector Laboratories, Burlingame, CA, USA), and anti-mouse CD44 (Abcam, Cambridge, MA, USA). Confocal scanning images were acquired on the LEICA DM2500 system (Leica Systems, Buffalo Grove, IL, USA) with either the 40×/1.15 Oil CS objective or the 63×/1.3 Oil CS objective. Immunohistochemistry (IHC) images of tissue sections were acquired on the Aperio AT Turbo slide scanner (Leica Systems, Buffalo Grove, IL, USA). To determine nuclear density, tumor cell nuclei were identified based on their unique morphology and the Nuclear v9 algorithm from Aperio was used to analyze the number of tumor cell nuclei per surface area (mm^2^). The entire tumor surface area was analyzed, excluding necrotic regions.

### Flow cytometry

Tumors were excised on day 20 post-inoculation and processed on GentleMACS Octo Dissociators using Mouse Tumor Dissociation Kits (both from Miltenyi Biotec, San Diego, CA, USA). Resulting single-cell suspensions were stained with LIVE/DEAD-Near IR viability dye (Thermo Fisher, Carlsbad, CA, USA) and Brilliant Violet 650-conjugated anti-mouse CD45 antibody (BioLegend, San Diego, CA, USA) according to manufacturer’s recommendations. Data were acquired on a Novocyte flow cytometer and analyzed using NovoExpress software (ACEA Biosciences, San Diego, CA, USA). GFP-expressing 3T3HAS3 fibroblasts were gated as live CD45^-^ GFP^+^ cells and quantified as a percentage of the CD45^-^ population (non-hematopoietic cells). For analyses of CD44 expression and HA binding of MDA-MB-468 shCD44 855 cells, cultured cells were dissociated with Accutase (BD Biosciences, San Jose, CA, USA) and stained with anti-mouse/human CD44 antibody IM7-phycoerythrin (eBioscience, San Diego, CA, USA) or sequential incubation with biotinylated HA [57] and streptavidin–phycoerythrin (SA–PE, R&D Systems, Minneapolis, MN, USA). CD44 expression and cell-associated HA levels in 3T3HAS3 CD44 KO cells were evaluated with the Alexa Fluor 488 anti-mouse/human CD44 antibody IM7 (BioLegend, San Diego, CA, USA) or sequential incubation with HTI-601 and SA–PE.

### Statistical analysis

Unpaired *t*-test (two tailed), one-way or two-way analysis of variance (ANOVA) were used for statistical analysis (GraphPad Prism 7.03, La Jolla, CA, USA). Tukey’s multiple comparisons test was used to compare between groups. Data were graphed as mean ± standard deviation (SD) or mean ± standard error of mean (SEM) as indicated in y-axis labeling.

## Abbreviations

CRISPR: clustered regularly interspaced short palindromic repeat
DMEM: Dulbecco’s Modified Eagle Medium
ECM: extracellular matrix
EGFR: endothelial growth factor receptor
FAP: fibroblast activation protein α
FGFR: fibroblast growth factor receptor
GFP: green fluorescent protein
HA: hyaluronan
HAS: HA synthase
HMW: high molecular weight
IHC: immunohistochemistry
KO: knock-out
MAPK: mitogen-activated protein kinase
PDGF: platelet-derived growth factor
PEGPH20: pegvorhyaluronidase alfa
PI3K: phosphoinositide-3-kinase
RPMI: Roswell Park Memorial Institute
SD: standard deviation
SEM: standard error of the mean
shRNA: small hairpin RNA
TME: tumor microenvironment
VEGFR: vascular endothelial growth factor receptor
